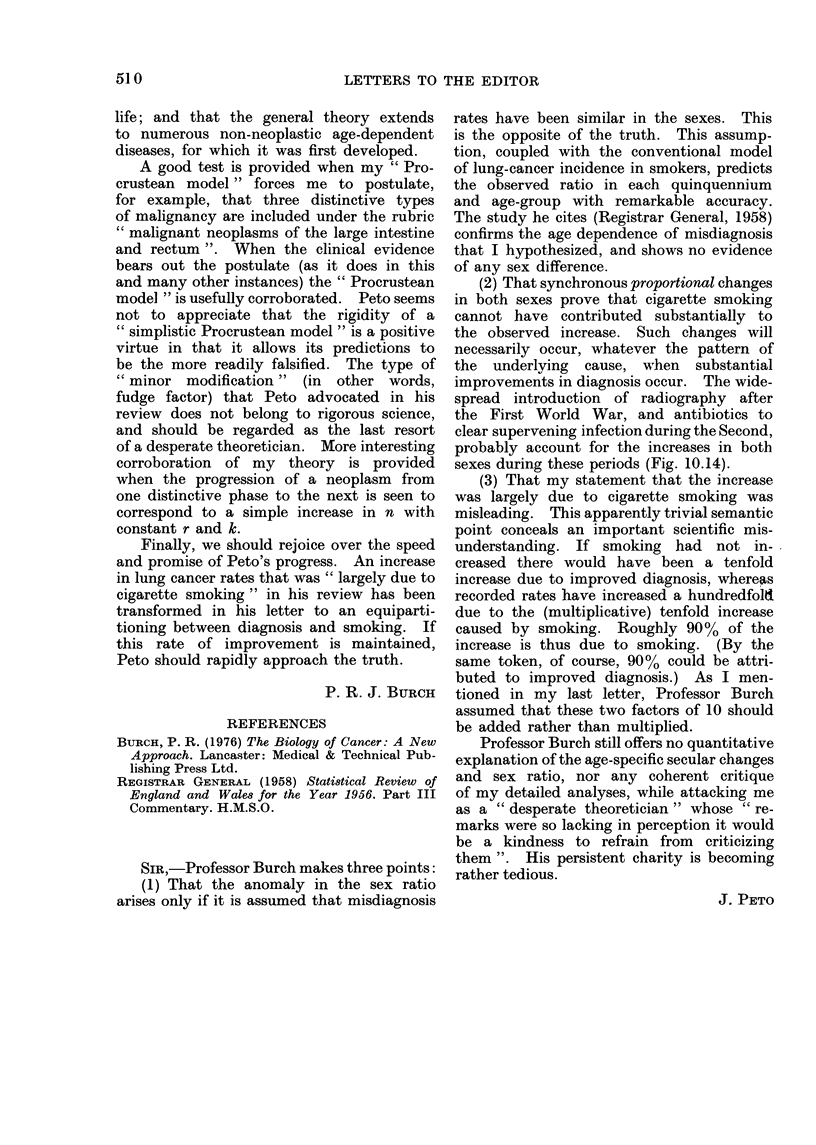# The Biology of Cancer—A New Approach

**Published:** 1977-04

**Authors:** J. Peto


Sir,—Professor Burch makes three points:

(1) That the anomaly in the sex ratio arises only if it is assumed that misdiagnosis rates have been similar in the sexes. This is the opposite of the truth. This assumption, coupled with the conventional model of lung-cancer incidence in smokers, predicts the observed ratio in each quinquennium and age-group with remarkable accuracy. The study he cites (Registrar General, 1958) confirms the age dependence of misdiagnosis that I hypothesized, and shows no evidence of any sex difference.

(2) That synchronous *proportional* changes in both sexes prove that cigarette smoking cannot have contributed substantially to the observed increase. Such changes will necessarily occur, whatever the pattern of the underlying cause, when substantial improvements in diagnosis occur. The widespread introduction of radiography after the First World War, and antibiotics to clear supervening infection during the Second, probably account for the increases in both sexes during these periods (Fig. 10.14).

(3) That my statement that the increase was largely due to cigarette smoking was misleading. This apparently trivial semantic point conceals an important scientific misunderstanding. If smoking had not increased there would have been a tenfold increase due to improved diagnosis, whereas recorded rates have increased a hundredfold due to the (multiplicative) tenfold increase caused by smoking. Roughly 90% of the increase is thus due to smoking. (By the same token, of course, 90% could be attributed to improved diagnosis.) As I mentioned in my last letter, Professor Burch assumed that these two factors of 10 should be added rather than multiplied.

Professor Burch still offers no quantitative explanation of the age-specific secular changes and sex ratio, nor any coherent critique of my detailed analyses, while attacking me as a " desperate theoretician " whose " remarks were so lacking in perception it would be a kindness to refrain from criticizing them ". His persistent charity is becoming rather tedious.

J. Peto